# The Pacific Medical and Surgical Journal

**Published:** 1858-06

**Authors:** 


*The Pacific Medical and Surgical Journal, edited by John B. Trask, M. D. and David Wooster, M. D.*

Yet another time is the experiment repeated of a Medical Journal in the land of gold, under the appropriate motto of *Iterum, Iteum, Iterumque.* In spite of the discouraging prospect which a retrospective view of California Medical Journals present, Drs. Trask and Wooster have ventured again into the field.

The first number of the Pacific Journal presents a creditable appearance, and contains much valuable matter. Dr. Toland, however, again lays claim to originality in his discovery of the reproduction of bones after the entire removal, referring especially to the phalanges of the fingers. His article is accompanied by an excellent illustrative lithograph. In the February number of this Journal, we took occasion to criticize Dr. Toland's claim to this discovery, quoting an article in the Transylvania Med. Journal, Dec., 1857, by Dr. Dudley,of Lexington,and an article by Dr. Hamilton, in this Journal, May, 1850. The practice recommended in these articles is identical with that employed by Dr. Toland, and, as in addition to the above-mentioned articles, an account of this practice appeared in the American Journal of Medical Science, we expressed surprise that Dr. Toland had not seen them.—Since then we have seen the account which we quoted, published in full in the Transactions of the American Medical Association, 1850, in the report of the standing committee on Surgery, page 352. In this article is given a full description of the practice of Prof. Dudley, with an account of cases by Prof. Hamilton, and a plaster cast of the parts. Though Dr. Toland has displayed much practical skill in his treatment of these cases, he should have been sufficiently familiar with the literature of the subject, to have prevented his laying claims to an original discovery. The real discoverer certainly cannot lie under the imputation of not having placed it prominently enough before the profession.

We wish the Pacific Journal every success, and from the appearance of the first number, are confident that it will not be undeserved.

